# *Campylobacter coli* Prosthetic Joint Infection: Case Report and a Review of the Literature

**DOI:** 10.3390/pathogens13100838

**Published:** 2024-09-27

**Authors:** Stijn Jonckheere, Celestin Mairesse, Patricia Vandecandelaere, Jens Vanbiervliet, Wim Terryn, Jan Somers, Benoit Prevost, Delphine Martiny

**Affiliations:** 1 Department of Laboratory Medicine, Jan Yperman Hospital, 8900 Ypres, Belgium; stijn.jonckheere@yperman.net (S.J.);; 2 Department of Microbiology, Laboratoire Hospitalier Universitaire de Bruxelles-Universitair Laboratorium Brussel (LHUB-ULB), Université Libre de Bruxelles (ULB), 1000 Brussels, Belgium; celestin.mairesse@lhub-ulb.be (C.M.);; 3 Department of Orthopaedic Surgery, Jan Yperman Hospital, 8900 Ypres, Belgium; 4 General Internal Medicine and Nephrology, Jan Yperman Hospital, 8900 Ypres, Belgium; 5 Faculty of Medicine and Pharmacy, University of Mons (UMONS), 7000 Mons, Belgium

**Keywords:** *Campylobacter coli*, prosthetic joint infection, WGS, virulome, antibacterial resistance

## Abstract

Prosthetic joint infections caused by *Campylobacter* are uncommon, with the majority of cases being attributed to *C. fetus*. This case report represents the third instance of a prosthetic hip infection caused by *C. coli* following an episode of gastroenteritis and, notably, in an immunocompetent patient. The infection was successfully managed by surgical debridement and lavage with retention of the prosthesis and 12 weeks of antibiotics. Furthermore, we present the first whole-genome sequence of a *Campylobacter* strain responsible for prosthetic joint infection and offer a comprehensive review of the literature on such infections.

## 1. Introduction

Campylobacteriosis is the leading bacterial cause of human gastroenteritis worldwide, and it represents a significant global public health problem. In 2022, there were 137,107 confirmed cases of human campylobacteriosis in the European Union, corresponding to a reporting rate of 43.1 cases per a population of 100,000 [1]. In the same year, the Foodborne Diseases Active Surveillance Network in the United States reported a campylobacter infection incidence of 19.2 cases per a population of 100,000 [2]. The exact burden of disease in low-resource settings, such as countries in Sub-Saharan Africa, is unknown due to limited surveillance, and the estimated prevalence of human infection in this region ranges from 8.5% to 14.1%, with prevalence rates reaching approximately 50% in children under 5 years of age and HIV patients [3].

As with other zoonoses, a significant decrease in the number of reported cases was observed in the 27 EU Member States during the pandemic, probably due to both underreporting and a real decrease in infections due to a reduction in international travel [2]. However, Kuhn et al. expect a significant increase in campylobacteriosis in the future due to global warming [4].

Poultry meat has been identified as a significant contributor to *Campylobacter* infections globally. However, other potential sources have been identified, such as other meats, raw milk, contaminated water supplies, or contact with pets [2,3]. *Campylobacter* infections can be prevented by practicing proper hand and cooking hygiene, consuming pasteurized milk or treated water, and taking precautions with animals, including pets.

The genus *Campylobacter* currently comprises 45 species and 13 subspecies, of which more than 10 are definitively associated with human infections. The filtration culture, which allows the isolation of non-thermophilic campylobacters, is currently limited to a few reference laboratories [5]. As a result, thermophilic *C. jejuni/coli* is considered the first bacterial cause of gastroenteritis worldwide and the most common zoonosis in Europe. There are significantly more *C. jejuni* than *C. coli*, and other *Campylobacter* species are probably greatly underestimated [5].

While gastroenteritis is a common manifestation of *Camplobacter* infection, focal extraintestinal infections occur uncommonly. To date, fewer than thirty cases of *Campylobacter* prosthetic joint infections have been reported worldwide, and they are predominantly caused by *C. fetus* in immunocompromised patients. This case study presents a case of *C. coli* prosthetic hip infection with bacteriemia following a gastroenteritis in an immunocompetent patient, the only relevant medical history of which was an uncomplicated gastric bypass. In addition, we provide a complete analysis of the strain’s whole-genome sequence and a comprehensive literature review, both of which should improve patient care in the future.

## 2. Case Description

A 61-year-old woman presented to the emergency department of Jan Yperman hospital (Ypres, Belgium) with atraumatic right hip pain, fever, and chills that had been ongoing for two days. She had a history of total hip prothesis 7 years prior and bariatric surgery (laparoscopic gastric bypass) 5 years prior. There was no nicotine abuse and only occasional alcohol consumption. Several weeks before her current onset of hip pain, she experienced anorexia and nausea. She had no direct contact with animals, had no recent travel, did not recall consuming undercooked meats, and had no ill contacts. The C-reactive protein (CRP) level was 177 mg/L, and she had a normal leukocyte count (5900/µL with 69.8% neutrophiles).

Based on the anamnestic and clinical findings, a prosthetic joint infection (PJI) was suspected, and a surgical debridement and lavage with synovectomy was performed on Day 2 of admission. During this procedure, a synovial punction and five tissue biopsies were taken. Besides antibiotic prophylaxis, no antibiotics were given before surgery, and vancomycin was intravenously initiated after surgery. The arthrocentesis of the right hip yielded 10 mL of bloody fluid, with 335,000 red blood cells/µL, only 11 leukocytes/µL, and an absence of crystals.

On Day 3 of admission, the aerobic blood culture bottles (BD BACTEC, BD Diagnostics, Sparks, MD, USA) taken on admission were flagged positive after 1 day, and 18 h of incubation and microscopic Gram-staining revealed Gram-negative rods. After culturing on blood agar, these were identified as *Campylobacter coli* via Matrix-Assisted Laser Desorption/Ionization-Time-of-Flight Mass Spectrometry (MALDI-TOF MS) using VITEK MS PRIME (bioMerieux, Marcy-l’Etoile, France) with knowledge base version 8.5.0-3. After two days of incubation, the hip punction and per-operative tissue samples all grew *C. coli*. Additionally, a feces sample that was taken on Day 3 of admission tested positive for PCR *Campylobacter* spp. using the Allplex GI-EB Screening Assay (Seegene, Seoul, Republic of Korea). Other targets were negative. The feces culture on Campylosel agar (bioMérieux SA, Marcy-l’Étoile, France) also revealed *C. coli*. Despite our best efforts, we were unable to identify any potential sources of the *C. coli* gastroenteritis. We also conducted a thorough review of the potential exposure to cattle or poultry but none were identified.

The antimicrobial susceptibility testing (AST) was conducted at the local laboratory and subsequently validated by the National Reference Center (NRC) through the utilization of gradient strips (Etest^®^, bioMérieux, Marcy l’Etoile, France) and the disk diffusion method, which was based on the EUCAST version 13.1 guidelines. The inoculum was applied to Mueller–Hinton agar supplemented with 5% defibrinated horse blood and 20 mg/L of β-NAD (MH-F), which was then incubated in a micro-aerobic environment at 41 ± 1 °C for 24 ± 1 h. When EUCAST breakpoints were not available, CA-SFM breakpoints 2023 v1.0 were used. An identical susceptibility profile was found for both the *C. coli* strains isolated from arthrocentesis and the feces culture. Based on the susceptibility results, antibiotic treatment was switched to levofloxacine 500 mg twice a day, which continued for 12 weeks.

Three days after the first debridement and lavage, a second extensive lavage was performed. The infected hip prothesis was retained. The patient made a good clinical and biochemical recovery with a favorable wound healing, and, after 10 days of admission, the patient was discharged from hospital with a CRP value of 15.1 mg/L. A full recovery was made with a pain-free hip joint and a CRP value of 3.2 mg/L one month after the discontinuation of antibiotics. Follow-up at 1 year showed no recurrence of infection.

Both strains were submitted to the NRC, which confirmed the phenotypic results (identification and AST) obtained by the local laboratory, and they were then submitted to whole-genome sequencing (WGS). DNA extraction was performed using an EZ1&2 Virus Mini Kit v2.0 on an EZ2^®^ Connect MDx instrument (Qiagen, Hilden, Germany). DNA was enzymatically fragmented according to the manufacturer’s instructions and modified to generate an Illumina-compatible DNA library using NEBNext^®^ Ultra™ II FS DNA Library Prep Kit for Illumina. The final libraries were qualified using an AATI Fragment Analyser (Agilent Technologies Inc., Santa Clara, CA, USA) with a DNF-474 High-Sensitivity NGS Fragment Analysis Kit, and they were then quantified using a Qubit 2.0 with a Qubit dsDNA HS Assay Kit (Life Technologies, Carlsbad, CA, USA). After equimolar pooling, the libraries were sequenced using a NovaSeq 6000 machine (Illumina Inc., San Diego, CA, USA) with a NovaSeq 6000 SP Reagent Kit v1.5 (300 cycles) in 2 × 150 bp paired mode. An average coverage of 100× was targeted.

FASTQ files from both sequenced genomes were processed using a Bionumerics v8.1 calculation engine (bioMérieux, Marcy l’Etoile, France). The de novo assembly was performed using the *SPAdes* genome assembler, and the quality of the assembly was assessed (Supplementary Materials, Table S1).

Multi Locus Sequence Typing (MLST) analysis classified both isolates as belonging to a new sequence type (ST-14004) (Supplementary Materials, Table S1).

Detection of the acquired resistance gene and point mutation was performed using Resfinder-4.5.0 for *C. coli*, with gene lengths of ≥80% and identities of ≥70%. Detection of virulence-associated genes was performed using the Custom Genotyping plug-in in BioNumerics v.8.1 based on the Basic Local Alignment Search Tool (BLAST, National Center for Biotechnology Information, US National Library of Medecine, Rockville Pike Bethesda, USA). The minimum sequence coverage and identity was set at 75%.

Table 1 provides a summary of the genes and point mutations observed in both strains, as well as those that were not identified but which were frequently considered to be reliable indicators of antimicrobial resistance [6]. Despite the absence of point mutations, seven resistant determinants were identified. The *blaOXA-460* gene, which is associated with resistance to beta-lactam antibiotics, was identified with 92.9% identity and 100% coverage. The *cmeB*, *cmeC*, *cmeD*, *cmeE*, and *cmeF* genes were identified with 100% coverage and 85%, 84%, 84%, 83%, and 85% identity, respectively. Additionally, *aadE-Cc* was identified with a coverage of 80% and an identity of 85%.

A total of 133 virulence genes associated with adhesion, immune modulation, motility, cytotoxicity, and invasion were screened, and 63 genes were identified in both isolates (Supplementary Materials, Table S1). Among these genes, 44 (70%) are known to be associated with bacterial motility, 16 (25%) with immune modulation, 2 (3%) with invasion into host cells, and 1 (1%) with adhesion (Supplementary Materials, Table S2).

## 3. Discussion and Review of the Literature

In this study, we reviewed the literature pertaining to *Campylobacter*-associated PJI. The review was restricted to articles published in English and referenced within the PubMed database. Twenty-six cases (including our case) have been reported [7,8,9,10,11,12,13,14,15,16,17,18,19,20,21,22,23,24] (Table 2). Most of these *Campylobacter* PJI were caused by *C. fetus* (n = 16), with only a few published cases of *C. jejuni* (n = 5), *C. coli* (n = 3), *C. lari* (n = 1), and *C. upsaliensis* (n = 1). There is no clear gender preponderance (58% being male) for *Campylobacter* PJI, with patients having a median age of 73 years (range: 24 to 88 years), and cases are equally distributed between the hip (n = 14) and knee (n = 12) PJI. There are no reports of *Campylobacter* PJI of the shoulder joint. Most published cases have been of immunocompromised patients (15 of 26 documented cases according to our interpretation); however, the patient in our case was not considered immunosuppressed as an uncomplicated gastric bypass was the only relevant medical history and there was no history of recurrent infections in the past.

Identical to the two previous case reports of *C. coli* PJI, our patient had a gastro-enteritis before the onset of the PJI, with a positive PCR and culture on stool. At the time of PJI diagnosis, blood cultures were also positive for *C. coli*, supporting the hematogenous seeding of the site of a prosthetic joint after a gastrointestinal illness. When considering the 26 published cases, 12 (46%) and 6 (23%) of them produced positive blood and stool cultures, respectively, although the results of these cultures were not obtained and/or not reported in all cases. Current treatment recommendations for *Campylobacter* gastro-enteritis is to limit treatment to those with severe disease or risk of severe disease. Patients with severe disease include individuals with bloody stools, high fever, extra-intestinal infection, worsening or relapsing symptoms, or symptoms lasting longer than one week [25]. Those at risk for severe disease include patients who are older, pregnant, or immunocompromised [26]. Based on the findings reported here and those found in our literature review, treatment might also be considered for those with prosthetic joints because patients with prosthetic joints may have a predilection to developing PJI after what may initially seem like an innocuous gastrointestinal illness. However, the impact on antimicrobial resistance and the potential side-effects of more widespread antibiotic use should be considered in relation to the potential prevention of a *Campylobacter* PJI, which is still a very rare occurrence.

A variety of surgical and antimicrobial regiments have been described for the management of *Campylobacter* PJI. Five cases have been managed without surgery; eleven with debridement, antibiotics, and implant retention (DAIR); and nine with one- or two-stage reimplantation arthroplasty. Various antimicrobial classes have been used, mostly in combination therapy and starting with an intravenous therapy followed by an oral phase, including ẞ-lactams (including ẞ-lactams + ẞ-lactamase inhibitors and carbapenems) in 60%, aminoglycosides in 44%, fluoroquinolones in 36%, macrolides in 32%, clindamycin in 20%, and tetracyclines in 8% of documented cases. The total duration of antibiotic treatment (for documented cases where the patient survived) ranged from 6 weeks to indefinite suppression therapy. Despite heterogeneous treatment, most cases have had a successful outcome even without surgical intervention. While these numbers are too small to draw conclusions, it seems that *Campylobacter* PJI’s have a favorable outcome without the need for an aggressive surgical approach or long-term antibiotic therapy. We successfully treated our patient as an acute Gram-negative PJI according to the recommendations of the PRO-IMPLANT foundation [27] with debridement and retention of the prosthesis, as well as 12 weeks of high dose fluoroquinolone after susceptibility was confirmed.

The WGS analysis enabled us to genetically characterize the two *C. coli* strains isolated from both the hip punction and fecal samples. The two strains showed an identical MLST allelic signature, identical resistance determinants, and identical virulence-associated genes (Supplementary Materials, Table S1).

No resistance genes known to be associated with macrolide and tetracycline resistance were identified, which is consistent with the susceptible phenotype (Table 1).

The *blaOXA-460* gene, a variant of *blaOXA-61*, was identified in both strains. These genes are associated with resistance to beta-lactams [28,29]. The identification of *blaOXA-460* was not found to be correlated with the phenotype (Table 1). The genotype–phenotype concordance of *blaOXA* genes was moderate, with a concordance of 67.4 observed for the *blaOXA-61* in the study by Wysok and colleagues [30].

The *cmeDEF* operon, which was also identified in both strains, has been described in *C. jejuni* as an efflux system with ampicillin as a substrate [31]. Nevertheless, we did not identify the *cmeDEF* operon in correlation with ampicillin resistance. The *cmeABC* operon codes for an active efflux pump has been associated with resistance to fluoroquinolone [32]. Absence of the *cmeA* gene in both strains suggested that the efflux pump complex might not form, and this would be consistent with the observed fluoroquinolone-susceptible phenotype (Table 1). Moreover, no point mutations associated with fluoroquinolone resistance were identified.

The *aadE-sat4-aphA3* gene cluster is associated with resistance to multiple aminoglycosides [33]. Both strains exhibited only part of this cluster, solely harboring the *aadE-Cc* gene. The lack of aminoglycoside resistance in these strains may be attributed to a truncated cluster, suggesting incomplete functionality.

Seventy percent of the genes identified in the virulence gene screening were genes encoding proteins that are responsible for bacterial motility associated with flagellar machinery (Supplementary Materials, Table S2) [34,35,36,37,38,39,40,41]. The flagellar machinery allows the bacterium to rotate and could be linked to adhesion and biofilm formation [42]. The flagellum in *Campylobacter* spp. plays a role in motility and is involved in several other biological processes, including secretion, colonization factors, cell division, and the regulation of these processes [42]. Screening for virulence factors identified 5 *kps* genes (*kpsD*, *E*, *M*, *S*, and *T*) associated with capsule biosynthesis and transport [43,44]. The deletion of the *kpsS-C-M-*gene in *C. jejuni* mutant strains has resulted in a notable reduction in their capacity to induce bacteremia in a murine model, suggesting a decreased infectivity for this bacterium [43,45,46,47]. Additionally, both *Cia*B and *Cia*C genes were identified. This evidence suggests that the bacteria possess the ability to secrete *Campylobacter* invasion antigens (Cia). The proteins in question may be transported by the flagellum and subsequently released into the cytosol of the host, where they may play a role in the organism’s virulence [48,49].

Our study will undoubtedly contribute to improving the diagnosis and management of patients suffering from PJI in the future. The literature review clearly demonstrated that *Campylobacter*, particularly *C. fetus*, is a potential cause of PJI. It should, therefore, form part of the diagnostic workup in clinical laboratories. This is crucial, given the specific growth conditions of the *Campylobacter* species (microaerobic atmosphere). Secondly, our literature review and case study definitively supports the optimal surgical and antibiotic approach for treating *Campylobacter* PJIs. Finally, the WGS analysis, when combined with more WGS data of invasive and non-invasive strains, will undoubtedly contribute to a better understanding of the pathogenesis of invasive infections in the future.

The primary limitation of our study is that only two strains from a single patient have been sequenced. Naturally, it is essential to sequence a substantial number of both invasive and non-invasive strains to obtain reliable conclusions.

## 4. Conclusions

*Campylobacter* prosthetic joint infections are a rare occurrence, they are predominantly caused by *C. fetus*, and they affect patients with immunocompromising conditions. Here, we reported a *C. coli* PJI in an immunocompetent patient.

Extensive surgical debridement with retention of the prosthesis in combination with antibiotics has been found, with an overall favorable prognosis, to be an efficacious treatment for such infections.

To date, no strain of *Campylobacter* responsible for prosthetic joint infections has been subjected to WGS as far as we are aware from an examination of the relevant literature. Our virulome analysis of this invasive *Campylobacter* strain identified several virulence factors. Further characterization of other invasive strains could reveal novel insight into the underlying mechanisms of the pathogenesis in *Campylobacter* strains involved in PJI and other invasive infections.

Consequently, it underscores the importance of systematically assigning invasive strains to the NRC.

## Figures and Tables

**Table 1:** Antimicrobial susceptibility testing and the genotypic antimicrobial resistance profile.

Antibiotic Class	Drug Tested	Disc Diffusion (mm)	Etest (mg/L)	S/I/R	Associated Resistance Gene(s) Found	Associated Resistance Gene(s) or Point Mutation(s) Not Found
Beta-lactams	AMP	30	1	S	*blaOXA-460* *cmeDEF*	*blaOXA-61* and *blaOXA*derivate ^a^
	AMC	38	0.125	S		
Quinolones	CIP	36	0.064	I	*cmeB*, *C*, *D*, *E*, *F*	Single- or multiple-point mutations (PM) in *gyrA.* *cmeABC* ^b^
Macrolides	ERY	24	1	S	None	*erm(B)*, *erm(N)*, PM in 23S_A2075G, 50S_L22_G86E
Tetracyclines	TET	35	0.25	S	None	*tet(O)*
Aminoglycosides	GEN	24	1	S	*aadE-Cc*	*ant(6)-Ia*, *aph(2″)-If*, *aph(2″)-Ig*, *aph(2″)-Ih*, *aph(2″)-IIIa*, *aph(3′)*, *aph(3′)-IIIa*, *aph(3′)-VIIa*, *aadE*, *aad9*, *sat4*. PM in *rpsL*_K43R, *rpsL*_K88R. *aadE-sat4-aphA-3* Gene Cluster

^a^ blaOXA-61, blaOXA-184, blaOXA-193, blaOXA-447, blaOXA-449, blaOXA-461, blaOXA-466, blaOXA-489, blaOXA-493, blaOXA-577, blaOXA-578, blaOXA-580, blaOXA-594, blaOXA-595, and blaOXA-603, blaOXA-623. ^b^ Operon cmeABC code for a multidrug efflux protein that contributes to resistance to different antimicrobial drugs. Abbreviations: AMP: ampicillin; AMC: amoxicillin–clavulanic acid; CIP: ciprofloxacin; ERY: erythromycin; TET: tetracyclin; and GEN: gentamycin.

**Table 2:** The Campylobacter prosthetic joint infections reported in the literature.

Year	Species	Age/Sex	Joint	Underlying Disease or Relevant Exposure	GI	BC	Stool	Joint Fluid/Periprosthetic Specimen	Surgery	Antibiotic Treatment	Recovery	Duration of Follow-Up	Ref.
1993	*C. fetus*	75/M	Hip	Chronic lymphocytic leukemia, prednisone	No	ND	ND	− (inhibited ^a^)	DAIR	IMI + GEN (7 d) AMO (LTAS)	Y	2 m	[7]
1994	*C. fetus*	68/F	Hip	rheumatoid arthritis, prednisone	No	ND	ND	+	DAIR	GEN + AZI (3 d) CHL (4 w)	DFUE	2 m	[8]
2003	*C. fetus*	76/M	Knee	None	No	+	ND	+	No surgery	CTR (6 w) CURa (9 m)	Y	10 m	[9]
2005	*C. fetus*	72/M	Knee	Cattle farmer	No	ND	ND	+	2SR	GEN + CIP + RIF (3 w) CIP (5 w)	Y	6 m	[10]
2005	*C. fetus*	72/M	Hip	Alcohol abuse, chronic granulocytic leukemia, hypertension	No	+	+	+	DAIR	CTR (3 w) ROX (3 m)	Y DFUE	2,5 y	[11]
2012	*C. fetus*	71/F	Knee	rheumatoid arthritis (corticosteroids + MTX), diabetes mellitus, cardiomyopathy	ND	ND	ND	ND	ND	CL +DOX (3 m)	Y	ND	[12]
2013	*C. fetus*	70/F	Knee	liver cirrhosis, alcohol abuse	ND	+	ND	+	1SR	AMC (6 w) CIP + CL (6 w)	DFUE	ND	[13]
2013	*C. fetus*	78/F	Hip	None	ND	−	ND	+	DAIR	CL (8 w) + GEN (2 w) AMO (LTAS)	Y	5 y	[13]
2013	*C. fetus*	88/M	Hip	Lung cancer	ND	+	−	+	No surgery	CL (4 w) + GEN (1 w) CL (LTAS)	Y	2 y	[13]
2013	*C. fetus*	85/F	Hip	Liver cirrhosis, alcohol abuse	ND	+	−	+	No surgery	AMO (4 w) + GEN (1 w) AMO (LTAS)	Y	3 y	[13]
2013	*C. fetus*	52/F	Knee	None	ND	−	+	+	1SR	AMC (6 w) + CL (1 w) CL (6 w)	Y	2 y	[13]
2013	*C. fetus*	76/F	Knee	None	ND	−	+	+	DAIR	CLR (4 w) + GEN (1 w) CLR (8 w)	Y	2 y	[13]
2013	*C. fetus*	75/M	Hip	Renal transplant	ND	+	+	+	1SR	CLR (4 w) + GEN (1 w) CLR (8 w)	Y	2 y	[13]
2016	*C. fetus*	77/F	Knee	ND	ND	ND	ND	+	DAIR	not specified (3 m)	Y	ND	[14]
2017	*C. fetus*	75/F	Knee	Diabetes mellitus, vascular disease	Yes	+	−	+	DAIR	MER (6 w) DOX (LTAS)	Y	4 m	[15]
2018	*C. fetus*	60/M	Hip	Diabetes mellitus, immunosuppressive disease, vascular disease	No	+	ND	+	2SR	IMI + AZI (6 w)	Y	ND	[16]
1993	*C. jejuni*	60/M	Hip	AIDS, B cell lymphoma, hemophilia A, liver cirrhosis	Yes	+	− (inhibited)	+	No surgery	GEN + CIP (17 d) ERY + CIP (8 w)	Y	2 m	[17]
2013	*C. jejuni*	77/M	Knee	immunosuppressive disease	ND	−	ND	9	1SR	AMC (6 w) + GEN (1 w) CIP (16 w)	Y	8 y	[13]
2013	*C. jejuni*	75/M	Knee	Thyroid cancer, hypertension	Yes	−	+	+ (PCR)	2SR	1/MER + AZI (6 w)-> AZI (7 w) 2/MER (2 w)->AZI (LTAS)	Y	3 m	[18]
2020	*C. jejuni*	73/M	Knee	Ulcerative colitis	No	+	ND	−	2SR ^b^	ETP (6 w) MOX + AMC (8 w)	Y	3 m	[19]
2022	*C. jejuni*	60 *F	Hip	Scleroderma (no immunosuppressive therapy), Raynaud’s phenomenon, chronic diarrhea	No	ND	ND	+	1SR	LEV (15/12 w before/after surgery)	Y	21 m	[20]
2009	*C. coli*	60/M	Hip	obesity, hypertension Ingestion of contaminated raw oysters	Yes	−	ND	+	DAIR	TIL (6 w) CIP (3 w)	Y	ND	[21]
2019	*C. coli*	63/M	Hip	Not immunocompromised Ingestion of undercooked chicken	Yes	−	ND	+	DAIR	CIP (6 m)	Y	6 m	[22]
2024	*C. coli*	61/F	Hip	Bariatric surgery	Yes	+	+	+	DAIR	LEV (12 w)	Y	1 y	
2002	*C. lari*	81/M	Hip	Cardiovascular disease not immunocompromised	No	+	ND	+	DAIR	PEN + FLU + GEN (2 d)	Death < sepsis at D2	ND	[23]
2002	*C. upsaliensis*	24/M	Knee	Osteoblastic osteosarcoma	Yes	−	− (inhibited)	+	No surgery	CTR + MET (2 w) CTR + ROX (4 w) AMO + ROX (6 m)	Y	34 m	[24]

^a^ Culture potentially inhibited by systemic antibiotics at the time of sampling. ^b^ Only one stage performed with retention of spacer. Abbreviations: GI: gastro-intestinal illness; BC: blood cultures; ND: not determined; DAIR: debridement, antibiotics and implant retention; LTAS: long-term suppression therapy; 1SR: one-stage reimplantation arthroplasty; 2SR: two-stage reimplantation arthroplasty; MTX: methotrexate; DFUE: death from unrelated event; AMO: antibiotics; AMC: amoxicillin, amoxicillin–clavulanic acid; AZI: azithromycin; CURa: cefuroxime axetil; CHL: chloramphenicol; CIP: ciprofloxacin; CTR: ceftriaxone; DOX: doxycycline; FLU: flucloxacillin; GEN: gentamycin; IMI: imipenem; LEV: levofloxacin; MER: meropenem; MET: metronidazole; MOX: moxifloxacin; PEN: penicillin; RIF: rifampin; ROX: roxithromycin; and TIL: ticarcillin–clavulanic acid. * a woman “in her sixties”.

## Data Availability

The original contributions presented in the study are included in the article/supplementary materials, further inquiries can be directed to the corresponding author.

## Supplementary Materials

The following supporting information can be downloaded at: https://www.mdpi.com/article/10.3390/pathogens13100838/s1, Table S1: The origin, host, source, isolation dates, MLST characteristics, genome metrics, antibioresistance, and the virulence gene detected in the two strains of Campylobacter coli; Table S2: The virulence-associated genes in both *C. coli* strains.
